# Conference Report: WORKSHOP ON PLANNING FOR COMPOUND SEMICONDUCTOR TECHNOLOGY Gaithersburg, MD February 3, 1995

**DOI:** 10.6028/jres.101.012

**Published:** 1996

**Authors:** Herbert S. Bennett

**Affiliations:** National Institute of Standards and Technology, Gaithersburg, MD 20899-0001

## 1. Introduction

This report describes the motivation for and the results of the Workshop on Planning for Compound Semiconductor Technology [1]. This Workshop, sponsored by the National Institute of Standards and Technology (NIST) and the Semiconductor Equipment and Materials International (SEMI), was held at Gaithersburg, Maryland on February 3, 1995, in conjunction with the International Workshop on Semiconductor Characterization: Present Status and Future Needs, January 30–February 2, 1995.

The purposes of the Workshop on Planning for Compound Semiconductor Technology were to:

1. Assess whether agreement exists in the compound semiconductor industry for the need of a consensus-based planning effort to support its future goals for materials, processes, devices, interconnects, and packages; and
2. Foster the free exchange of information and ideas that might be used to create a more competitive compound semiconductor industry by a mutual understanding of its common problems and of ways to solve them.

Without some consensus-based planning on the part of the North American compound semiconductor industry, future economic opportunities in this industry may be limited.

## 2. Motivation

Over the past decade, the North American share of world markets for products based on compound semiconductors has declined [2]. To reverse this trend in the North American market share of products containing compound semiconductors, some consensus-based planning may be worthwhile in view of the limited resources available to this industry for building its supporting infrastructure because:

1. Very strong growth is expected in the worldwide compound semiconductor market in large part due to the exploding information technology industry [3] and
2. The compound semiconductor industry is much more diverse and fragmented than the silicon semiconductor industry.

Many optoelectronic components contain compound semiconductor devices. One of the reasons for the formation of the Optoelectronics Industry Development Association was to respond to the loss in market share of optoelectronics produced in the United States. In 1980, most of the optoelectronics consumed in the world came from the United States. By the end of the decade, 70 % came from Japan [4]. The Japanese share is larger than 70 % in some product categories for optoelectronics. For example, even though prototype flat-panel liquid-crystal displays were developed in the United States, Japan now has about 95 % of the global market for flat-panel displays, primarily, active matrix liquid displays. Semiconductor Equipment and Materials International (SEMI) estimates that the global flat-panel display market will be about $7 billion in 1995 [5].

Attendees at the recent International Workshop on Semiconductor Characterization discussed the technical opportunities for both silicon and compound semiconductor technologies [6, 7]. The merging of computer, television, and communication-network systems offers many unique applications for compound semiconductors. Such applications include microwave devices, light-emitting diodes, laser diodes, flat-panel displays, detectors, sensors, and very high-speed electronics. Such compound semiconductor devices will be used in systems for wireless communications, optical communications, very high-speed networks, and imaging. All of these systems are critical for future information technologies.

Among the many compound semiconductor technologies, GaAs integrated circuits (ICs) technologies have received above-average attention from firms that do market analyses. Four market research firms have recently prepared reports on their expectations of the GaAs IC market by the year 2000 [8]. These four firms are Integrated Circuits Engineering, Kenneth W. Taylor and Associates, Electronic Trend Publications, and The Information Network. A common theme in these reports is that GaAs ICs will be used for high-volume, consumer-driven applications and that the growth rate in the global GaAs IC industry will be very positive. Selected highlights from Ref. [8], based on reports from the above-mentioned firms, are given in the next four paragraphs.

Integrated Circuits Engineering (ICE) predicts that the worldwide GaAs IC market will increase from its expected $535 million in 1995 to greater than $1 billion in 1998. About 75 % of this production will come from 10 firms of which 5 are United States based. ICE calculates a 23 % compound annual growth rate for the GaAs IC industry between 1994 and 2000. Much of this growth will be for analog circuits operating at the 2 GHz and higher frequencies for wireless communications. ICE also predicts a declining role for military and aerospace applications of GaAs ICs. But this decline will be more than offset by the increases in commercial applications such as telecommunications.

Kenneth W. Taylor and Associates (KWTA) believes that digital wireless communications are going to be the big growth sector for the GaAs industry. Including both merchant and captive digital GaAs production, KWTA says that this sector should double this year from $72 million in 1994 to $177 million in 1995 and probably should reach $2.2 billion by the year 2000. The digital wireless communications market will be dominated by planar structures. But, by the year 2000, heterostructure circuits will most likely represent 30 % of the global digital wireless communications market. KWTA also predicts that the number of digital wireless communications terminals will increase from 34.5 million units in 1994 to 585 million units by 2000. This growth rate is much greater than that for television receivers. For comparison, it is estimated that there are about 500 million television receivers today, and it took over 45 years to achieve that number. The largest applications for digital wireless communications terminals are likely to be radio frequency (rf) transponders for security, smart highways, toll collection, and industrial process controls.

Electronic Trend Publications has examined the semiconductor content of mobile communications products such as pagers, cellular phones, cordless phones, and the like. The market for all RF/Intermediate Frequency (IF) circuits, both GaAs and Si, is expected to increase to $1.7 billion in 2000 from $611 million in 1993. However, the GaAs share of the RF/IF market depends on many technical factors such as total cost of manufacturing, size, reliability, and power consumption. The GaAs share also depends on nontechnical factors such as experience and prejudices of designers as well as the fabrication capabilities and management directions taken by companies producing RF/IF circuits. GaAs excels in performance for power amplifiers. The market for GaAs monolithic microwave integrated circuits (MMICs), which includes power amplifiers and transmit/receive switches since they have more than one transistor, is expected to grow from $75 million in 1995 to about $600 million by 2000. The latter would be about a 62 % market share with silicon.

The Information Network analyzed merchant and captive markets for digital GaAs devices, and predicted a compound annual growth rate of about 22 % from $123 million in 1994 to $333 million in 1999. The digital GaAs market is driven by fiber optic telecommunications, high-speed data networking, and high-end supercomputers, workstations, and general purpose computers. The anticipated growth for analog/microwave GaAs ICs, again both merchant and captive markets, is from $292 million in 1994 to $919 million in 1999. According to the Information Network, the United States should continue its 51 % share to 1999. Military applications used to account for the bulk of the U.S. market. But, that has shrunk to 25 % and will continue to decrease. The driving forces in the United States for increased use of GaAs ICs are applications for long-haul telecommunications over fiber, wireless that includes cellular and set-top boxes, local area networks, identification tags, toll collection, ground position satellites, and direct broadcast satellites. Communications and consumer applications will drive the GaAs markets in Japan and Europe. Due to the early emergence of the U.S. GaAs market, its GaAs market probably will grow slightly slower at 22 % as compared to the Japanese and European market at 27 % during the period covered.

In order to place the above market analyses for GaAs ICs in perspective, we should consider some data presented by Roland Haitz in his talk on “Visible Light Source Applications.” The top six competitors in making semiconductor-based optoelectronic components, excluding liquid-crystal displays, have a combined 1994 estimated revenue from those components of about $2 billion. Individually, the present global markets for semiconductor-based optoelectronics and for flat-panel displays are much larger, by more than a factor of 3, than the present global market for GaAs ICs. The above-mentioned 51 % share for the United States in the global $500 million GaAs IC market is good; but it has to be viewed in the context of a 27 % share in the global $2 billion semiconductor-based optoelectronics market and of less than a 5 % share in the global $7 billion flat-panel display market.

## 3. Workshop Highlights

### 3.1 Results

The Workshop was attended by approximately 60 people from industry, government, and academia. It consisted of relevant invited talks and discussions by experts knowledgeable in the field of compound semiconductor technology and of commercial markets that rely on this technology to meet customer demands.

The Workshop attendees agreed that a consensus on the need for such planning does exist, and that if such planning occurs, it is more appropriate to use existing industry and government organizations such as those listed below. The attendees also proposed future actions such as:

1. Form an industrial alliance on planning for compound semiconductors consisting, as appropriate, of such organizations as the Optoelectronics Industry Development Association (OIDA), Microwave Solid State Electronics Division (MSSED) of the Electronics Industry Association (EIA), Semiconductor Industry Association (SIA), and the Semiconductor Research Corporation (SRC), as appropriate.
2. Coordinate the activities of the proposed compound semiconductor alliance with related activities in such agencies as the Advanced Research Projects Agency (ARPA), the National Institute of Standards and Technology, and the National Science Foundation and other interested parties such as those contributing to the National Electronics Manufacturing Initiative.
3. Determine to what extent the above-mentioned U.S. Government agencies and other interested parties are able to provide funds and/or staff to form the compound semiconductor alliance, to organize the first few meetings, to facilitate industry leadership, and to reimburse travel expenses of invited participants at alliance meetings.
4. Form a parallel organization for the microwave/radio frequency industry that addresses questions similar to those addressed by OIDA for optoelectronics.

These recommendations for action represent some of the highlights and main ideas expressed by the invited speakers and attendees and summarized the major conclusions reached by them. The inclusion of a recommendation here does not imply that it was universally accepted by all Workshop participants, but rather that it was expressed by several of the speakers and participants. The above recommendations are based on those attending the Workshop, and their views may not be representative of those in the compound semiconductor industry as a whole.

Any consensus-based planning for compound semiconductors should try to have inputs and advice from those involved with planning for silicon semiconductors. There are both non-technical and technical reasons for why those who planned for silicon should be asked to advise in the planning for compound semiconductors. Those who contributed to the National Technology Roadmap for Semiconductors (NTRS) [9] have considerable experience in developing and implementing consensus-based efforts that are led by industry and can provide the compound semiconductor planners with lessons learned from the NTRS, which addresses silicon integrated circuits for primarily memory and microprocessor applications. Present forms of GaAs very large-scale integration (VLSI) circuits are higher performing versions of silicon VLSI circuits [10]. Both kinds of integrated circuits are based on similar circuit concepts and have related technical challenges in interconnections and packaging. The equipment used to make present GaAs VLSI circuits is based substantially on manufacturing technology and equipment for making silicon ICs. Also, the future techniques for performing the functions of interconnecting and packaging silicon ICs could very well involve compound semiconductors [11].

The attendees did not reach an agreement on which applications among the numerous existing or potential applications of compound semiconductors are most opportune for planning efforts. Nor did they reach an agreement on an algorithm for deciding how to focus planning for such a broad area as compound semiconductors. These latter two issues–which applications and how to focus the proposed plan–will have to be considered in future activities on planning for compound semiconductors. Some of the speakers and participants presented strong cases that a successful plan might concentrate on:

1. Blue-light sources based perhaps on GaN materials for displays, optical memory, hard opy, and “white” illumination, and
2. High-speed, electronic circuits for wireless and optical communications that include microwave, digital, and mixed signal circuits and that are based on GaAs materials.

Such a plan could include material systems, process integration, device design, packaging, and computer modeling.

An overwhelming majority of the approximate 60 attendees indicated that they would be willing to participate in an appropriate alliance to develop a plan for compound semiconductors.

### 3.2 Questions Addressed

The Workshop addressed the question: Does the compound semiconductor industry need a strategic plan for selected aspects of its goals in materials, processes, devices, interconnects, and packages?

Other questions requiring answers in order to build a consensus for action were discussed. These included:

- In the context of planning for compound semiconductors, what should compound semiconductors include? GaAs, InP,GaN, SiC, SiGe, or others?
- In what form should this plan evolve?
- Who will sponsor the development and maintenance of the plan?
- Who will provide the resources?
- How will this plan relate to the other planning activities such as the Optoelectronics Industry Development Association (OIDA) Roadmap [12] and the National Electronics Manufacturing Initiative (NEMI)? [13] What is the role for other organizations such as the Lasers and Electro-Optics Manufacturing Association?
- Who speaks for compound semiconductors?
- Who are our customers and stakeholders?

Three examples of ongoing consensus-based planning are:

1. The National Technology Roadmap For Semiconductors [9] that addresses the needs of silicon-based digital ICs for primarily memory applications and microprocessors;
2. The Optoelectronic Technology Roadmap [12] that addresses the needs of optoelectronic technologies for display, optical storage, optical communication, and hardcopy applications; and
3. The Electronics Manufacturing Technology Roadmaps [14] for electronic interconnection substrates, radio frequency communications, photonics, packaging, board assembly, precision electromechanical assembly, electronics manufacturing equipment and process, and rapid physical and virtual prototyping.

These planning activities are industry-led, with government participation and facilitation. The attendees agreed that the proposed planning for compound semiconductors should also be led by industry, with government participation and facilitation.

### 3.3 Organization

The Workshop had three sessions:

- Commercial/Industrial Market Applications for Compound Semiconductors–The Technology Drivers
- Status of Related Compound Semiconductor Activities
- Panel Discussion–Should the Compound Semiconductor Industry Speak in Unison about Its Future? And, if so, how would this be accomplished?

Due to the time limitations of a 1-day workshop, not all aspects of compound semiconductors were addressed. By necessity, Session I addressed a subset of the broad area of compound semiconductors:
1.Wireless Applications*Walter Davis*, Corporate Vice President and Director of Strategic Semi-conductor Operations, Motorola2.Visible Light Source Applications*Roland Haitz*, Group Research and Development Manager, Hewlett-Packard3.Opto-Electronics for Lightwave Communication Systems*Young-Kai Chen*, Department Head for High-Speed Electronics and Devices, AT&T Bell Laboratories

The above three invited speakers for this Session devoted portions of their presentations to those markets, today and future, for which compound semiconductors are expected to be more competitive than alternative semiconductors such as silicon. They considered market applications and technical performance requirements of systems. Namely, what do the users want? With that information on expected systems performance, they presented examples of those requirements or specifications that are likely to be satisfied better by compound semiconductors than by other alternatives. They concluded their talks by answering from their perspective subsets of the previously mentioned questions “a” through “g”

Session II addressed other ongoing consensus-based planning activities and contained examples on the role of government. This session contained three talks on:
1.OIDA Planning Activities*Roland Haitz*, Group Research and Manager, Hewlett-Packard, and OIDA Board Member2.Advanced Research Projects Agency (ARPA) Programs and Thrusts in Compound Semiconductor Technologies*Sven Roosild*, Deputy Director, Microelectronics Technology Office, ARPA3.National Electronics Manufacturing Initiative*Herbert Bennett*, Senior Research Scientist, NIST

Session III was a panel discussion on whether the compound semiconductor industry should speak in unison about its future, and if it should, how this would be accomplished. Herbert Bennett was the moderator, and the panel members were Walter Davis, Roland Haitz, Young-Kai Chen, and Sven Roosild. The Workshop attendees concluded that:

1. The compound semiconductor industry consists of three major industry segments: optoelectronics, microwave circuits, and electronic circuits.
2. Existing industrial organizations and the government should collaborate to conduct the proposed planning.
3. No one single, recognized, organization speaks for compound semiconductors.
4. Flexible, intelligent manufacturing with real-time feedback control of the process steps offers great opportunities for compound semiconductors.
5. It would be better to use existing organizations to form an alliance to plan for compound semiconductor technologies.

Such an alliance might be composed in part of the OIDA, the Microwave Solid State Electronics Division (MSSED) of the Electronics Industry Association (EIA), and perhaps the Semiconductor Industry Association (SIA), and/or the Semiconductor Research Corporation (SRC). The OIDA would represent optoelectronics, the MSSED/EIA would represent monolithic microwave circuits, and the EIA and perhaps the SIA-SRC combination would represent the digital and analog integrated circuits. Such an alliance would sponsor the development of the plan, maintain the plan, provide the necessary resources, and speak for the compound semiconductor industry.

The Workshop was organized by a Workshop Committee chaired by *Herbert S. Bennett*, National Institute of Standards and Technology. Other members of the Workshop Committee were *Jimi Dixon*, Semiconductor Equipment and Materials International; *Paul Amirtharaj*, NIST; *Frank Oettinger*, NIST; and *David G. Seiler*, NIST.

Reference [1] contains edited summaries of the speakers’ answers to the foregoing seven questions, “a” to “g,” the questions and answers that followed each talk given in Sessions I and II, and the edited highlights of the panel discussion in Session III. Its appendices have an attendees list, summaries of some industry-Government activities in compound semiconductors for microwave and millimeter-wave integrated circuits, and copies of the vugraphs used by the invited speakers.

The terms “roadmap,” “plan,” and “strategic” plan occurred frequently during the Workshop’s discussion. Because these terms have not been adequately discussed in the context of the compound semiconductor industry, it is best for the purposes here to consider them as not defined precisely. However, any coordinated planning for compound semiconductors will probably be more extensive in the number of technologies considered and be more globally based than the well-focused National Technology Roadmap for Semiconductors that is primarily limited to the CMOS technology needs of digital integrated circuits for memory and logic products.

### 3.4 Salient Excerpts From Talks

#### Wireless Applications

##### Walter Davis, Motorola

From the customer perspective, the primary focus that we see is on gallium arsenide, mainly because it is closer to maturity in markets. The prototype of success for industry and government cooperation has been SEMATECH. SEMATECH is a good example of how to do cooperative, consensus-based planning. We are moving from an era of performance-driven designs to one in which power drain will be the most important attribute.

#### Visible Light Source Applications

##### Roland Haitz, Hewlett-Packard

Compound semiconductors should include all group III–V and II–VI material systems that are used to make pn junction devices. Also, GaN, ZnSe and HgCdTe should be included. Issues for the proposed plan are technology support needs, manufacturing processes, infrastructure for equipment and material suppliers, and coordination of government-funded research.

#### Opto-Electronics for Lightwave Communication Systems

##### Young-Kai Chen, AT&T Bell Laboratories

As fiber networks are deployed to neighborhoods, more and more compound semiconductors devices will be needed. In the 1980s, you could afford to spend $1000 to $10,000 for a module in a long distance trunk that was shared by many, many users. But, in the local loop as fewer people share those costs, the costs must decrease to less than $200.

#### OIDA Planning Activities

##### Roland Haitz, OIDA Board of Directors

One of the OIDA’s findings is that the United States is far behind Japan in making high volumes of flat-panel displays with high information content. Even if the migration of electronics and optoelectronics to the back side of displays does not occur, the U.S. optoelectronics industry still must regain its lost market share by producing high-volume products that contain integrated electronics and optoelectronics components.

#### Advanced Research Projects Agency (ARPA) Programs and Thrusts in Compound Semiconductor Technology

##### Sven Roosild, ARPA

ARPA supports areas for which silicon cannot do the job such as components involving visible and infrared light emission, very high speed, and those advanced nanoelectronic devices made from compound semiconductors. When the conventional decrease in linewidths and reduced design rules are no longer physically possible, we clearly have to fabricate new devices that today are most likely to involve compound semiconductors.

#### National Electronics Manufacturing Initiative

##### Herbert Bennett, NIST

If the Clinton Administration’s ongoing initiatives concerning the National Information Infrastructure are successful, they will create considerable demands for high-volume, low-cost electronics hardware to implement them. For this reason, it became appropriate in 1993 to complement these initiatives with a National Electronics Manufacturing Initiative, led by industry, to promote the manufacture of information and consumer electronics products.
